# Ortner's Syndrome (Cardiovocal Syndrome): A Case Report

**DOI:** 10.7759/cureus.38408

**Published:** 2023-05-01

**Authors:** Pablo Arango Guerra, Carolaine Ortega-Agamez, Sebastian Naranjo-Restrepo

**Affiliations:** 1 Internal Medicine, Universidad CES, Medellin, COL; 2 Internal Medicine, University of Cartagena, Cartagena, COL; 3 Cardiology, Clinica CES, Medellin, COL

**Keywords:** heart failure, vocal fold paralysis, clinical cardiology, hoarse voice, ortner syndrome

## Abstract

Ortner's syndrome, also known as cardiovocal syndrome, refers to vocal cord paralysis caused by an underlying cardiovascular condition. It is often due to the constriction of the left recurrent laryngeal nerve by the pulmonary artery or left atrium. Recurrent aspiration pneumonia is a frequent complication, which can result in substantial morbidity and mortality. Early recognition and treatment, as well as the resolution of the underlying cause, when feasible, can enhance the otherwise unfavorable prognosis of this condition. In this particular case, a 65-year-old man with idiopathic dilated cardiomyopathy was diagnosed with hoarseness and evidence of left vocal cord palsy.

## Introduction

Ortner's syndrome, also called cardiovocal syndrome, is a form of vocal cord paralysis caused by a cardiovascular issue. Norbert Ortner was the first to describe it in 1897 as a paralysis of the left vocal cord resulting from mitral stenosis. He believed that the enlargement of the left atrium was responsible for compressing the recurrent laryngeal nerve. However, it has since been described in various cardiovascular conditions including valvular heart disease and pulmonary hypertension that result in the dilatation of the heart chambers and compression of the left recurrent laryngeal nerve [1-3]. We present a case of a patient with Ortner's syndrome secondary to idiopathic dilated cardiomyopathy.

## Case presentation

**Figure 1:**
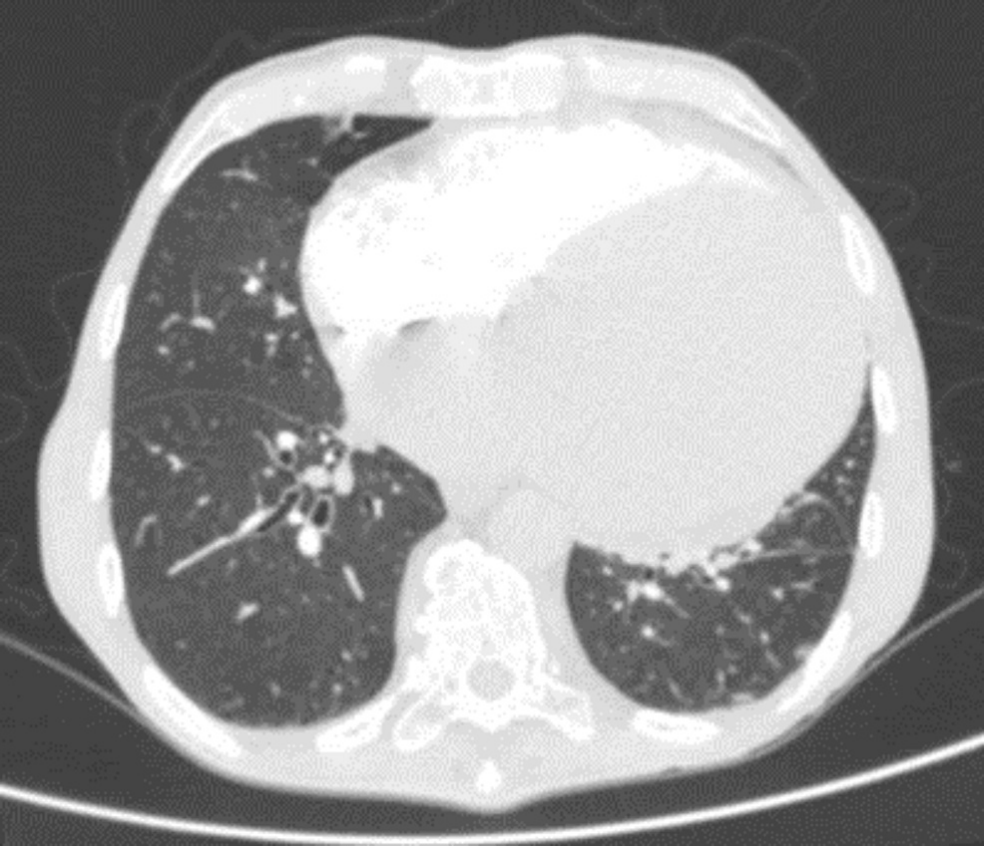
Severe dilatation of the cardiac chambers, predominantly on the left.

This is a 65-year-old male patient without previous known medical history, who was admitted due to dyspnea and bilateral leg edema for approximately two weeks. Additionally, he reported incipient dysphonia which was also noted by his family. Multiple studies were performed during the hospital stay including electrocardiogram, chest radiography, and echocardiography revealing dilated heart disease with severe biauricular and biventricular dilation (Figure 1) and an ejection fraction of 10%, leading to a diagnosis of de novo heart failure.

After an extensive evaluation and after ruling out ischemic, metabolic, and arrhythmogenic etiology, it was decided to perform a cardiac MRI, which did not show areas of fibrosis, late gadolinium enhancement, and other findings suggesting sequelae of myocarditis, infiltrative pathology (amyloidosis, iron overload) or sarcoidosis. Therefore, it was concluded that it was an idiopathic dilated cardiomyopathy.

**Figure 2:**
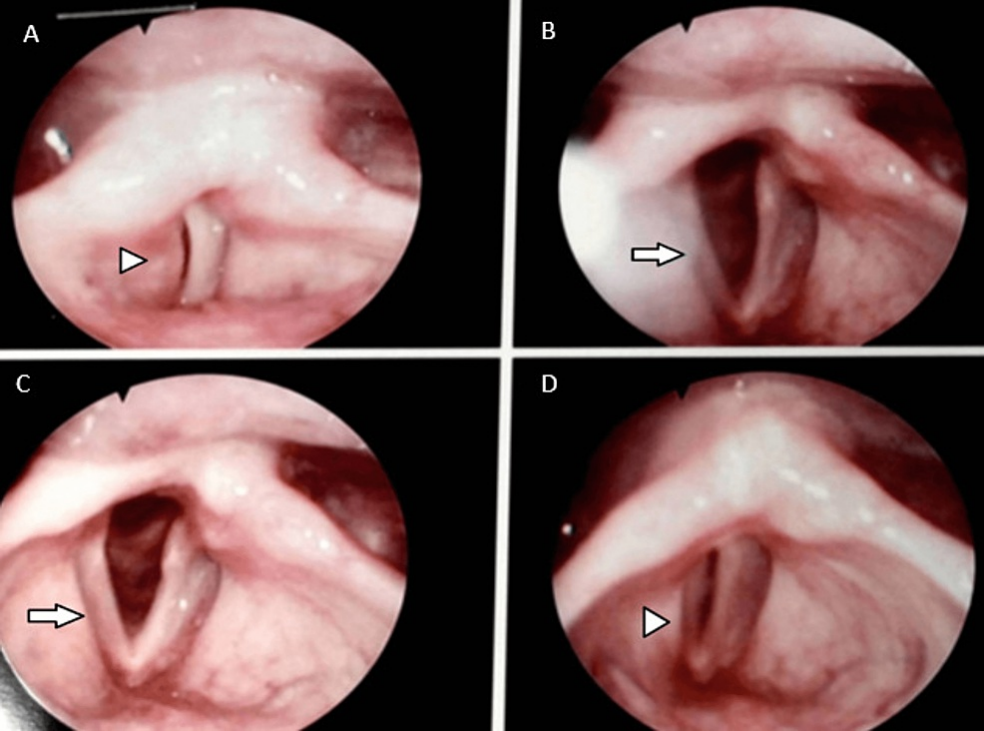
A-D: Incomplete glottic closure with hypotrophic and bowed left vocal cord (arrowhead). B-C: Abduction of the vocal cords, with hypotrophic and arched left vocal cord (arrow). This indicates left vocal cord paralysis.

During the hospitalization, the patient's dysphonia worsened, leading to an evaluation by an otolaryngology (ENT) specialist. A nasofibrolaryngoscopy was performed, which revealed left vocal cord paralysis (Figure 2) without any signs of intrinsic laryngeal pathology or tumor.

Therefore, a tomography of the neck and thorax was performed in order to rule out compressive tumor pathology; however, this was not evidenced, and it was determined that the cause of the left vocal cord paralysis was secondary to extrinsic compression of the left recurrent laryngeal nerve since the cardiomegaly which generated a displacement of the different mediastinal structures was evident. Due to the above and in conjunction with ENT, it was concluded that it was Ortner's syndrome. Since the patient did not present episodes of bronchial aspiration, it was decided to perform intensive medical management consisting of optimizing the therapy of his cardiomyopathy. Unfortunately, the patient's follow-up was lost, and we have no information after the diagnosis and subsequent outcomes.

## Discussion

The prospective study by Loughran et al. found that left recurrent laryngeal nerve palsy is more frequent than the right, with a proportion of 67% and 33% of cases, explained by the longer and deeper course of the left recurrent laryngeal nerve within the thorax, since this emerges from the aortic arch passing then through the aortopulmonary window. This leads to the left one being more susceptible to extrinsic compression by multiple causes, unlike the right one which emerges from the right brachycephalic bifurcation with a much shorter mediastinal course. It can occur at any age, with a higher incidence in men, with malignant tumor pathology being the most common cause (52%) of extrinsic compression and lung and esophageal cancer the main ones, while other causes such as iatrogenic, post-traumatic, and idiopathic are found in 22%, 5%, and 11% of cases, respectively. Ortner's syndrome makes up less than 11% of cases, and in other studies, the proportion is lower, ranging from 1.5% to 6.3% [4,5]. It is important to note that recurrent laryngeal nerve compression occurs indirectly, with the left pulmonary artery displaced upward toward the aorta, erasing the aortopulmonary window and resulting in nerve compression [6,7]. Although rare, other cardiac conditions, including pulmonary hypertension, aneurysms of the left ventricle or aorta, and congenital heart disease, can lead to morphological changes in the cardiac chambers and carry the risk of nerve damage due to the same mechanism of effacement of the aortopulmonary window mentioned previously [8-11].

Symptoms of vocal cord paralysis consist of hoarseness or dysphonia, difficulty swallowing or dysphagia, and trouble breathing when speaking due to air leakage caused by glottic incompetence. Patients also have an ineffective cough, and there is a high risk of aspiration, particularly with liquids [2]. These symptoms may overlap with those caused by heart failure, and sometimes they may be misdiagnosed as heart failure, particularly if it's not known that vocal cord paralysis can be due to cardiovascular issues. It's important to note that this condition is not always permanent. Beyond recognizing cardiovascular pathology as a cause of vocal cord paralysis, it is more important to emphasize the idea that, although a plausible explanation may exist for this condition, it is more relevant to first rule out other malignant and non-malignant tumor pathologies, such as cervical, thoracic, and mediastinal, as well as intrinsic laryngeal pathology, by always performing laryngoscopy, chest X-ray, neck and chest tomography, and endoscopy, thus addressing all potential causes before determining that it is Ortner's syndrome. Early identification and treatment of the underlying cardiovascular cause can be corrected and can have a positive impact on the patient's quality of life. Moreover, it could be an initial sign of a potentially deadly vascular disease, and prompt medical investigation is necessary [1,12,13].

When the underlying cause cannot be corrected, two absolute reasons for endoscopic surgical intervention are aspiration pneumonia and the patient's desire to improve their voice quality. Medialization of the paralyzed vocal cord decreases the risk of aspiration pneumonia and enhances the sound quality of the patient's voice [14,15].

## Conclusions

Based on the above description, the aim of this case report is to increase awareness and enhance the understanding of clinical features, especially in patients with heart disease, recognizing that comorbidities go beyond metabolic ones and also include others such as Ortner's syndrome, which, although a relatively uncommon condition, can be considered an extra-cardiac manifestation with the potential to be very symptomatic and produce complications. Its recognition provides an explanation for specific symptoms, and early identification can help prevent secondary complications. Therefore, the importance of interdisciplinary treatment with the ENT specialist should be highlighted.
